# Differential Expression of Galectin-1 and Galectin-9 in Immune-Mediated Inflammatory Diseases

**DOI:** 10.3390/ijms26189087

**Published:** 2025-09-18

**Authors:** Cristina Valero-Martínez, Marisa Pardines-Ortiz, Nuria Montes, Esteban Dauden, Benjamín Fernández-Gutierrez, Esther García-Planella, Fernando Gomollón García, Jordi Gratacós, Jose Javier Pérez-Venegas, Antonio Julía, Sara Marsal, Amalia Lamana, Rosario García-Vicuña, Isidoro González-Alvaro, Ana Triguero-Martínez

**Affiliations:** 1 Rheumatology Department, Hospital Universitario La Princesa, Instituto de Investigación Sanitaria La Princesa (IIS-Princesa), 28006 Madrid, Spain; cristina.valmart@gmail.com (C.V.-M.); marisavilladebiar@hotmail.com (M.P.-O.); nmontes@salud.madrid.org (N.M.); amaliala@ucm.es (A.L.); vicuna111@gmail.com (R.G.-V.); 2 Dermatology Department, Hospital Universitario La Princesa, Instituto de Investigación Sanitaria La Princesa (IIS-Princesa), 28006 Madrid, Spain; estebandauden@gmail.com; 3 Rheumatology Department, Hospital Clínico San Carlos, 28040 Madrid, Spain; benjamin.fernandez@salud.madrid.org; 4 Gastroenterology Department, Hospital de la Santa Creu i Sant Pau, 08041 Barcelona, Spain; egarciapl@santpau.es; 5 Gastroenterology Department, Hospital Clínico Universitario Lozano Blesa IIS Aragón, CIBEREHD, 50009 Zaragoza, Spain; fgomollon@gmail.com; 6 Department of Rheumatology, Parc Tauli Hospital Universitari, I3PT-CERCA, Medicina UAB, 08208 Sabadell, Spain; jgratacos@cspt.es; 7 Rheumatology Service, Hospital Universitario Virgen Macarena, 41009 Sevilla, Spain; perez.venegas@gmail.com; 8 Rheumatology Department, Hospital Universitari Vall d’Hebron, 08035 Barcelona, Spain; toni.julia@vhir.org (A.J.); sara.marsal@vhir.org (S.M.)

**Keywords:** immune-mediated inflammatory diseases, Galectin-1, Galectin-9, biomarkers

## Abstract

Galectin-1 and -9 (Gal1/9) are essential mediators of immune-inflammatory responses, which makes these proteins potential biomarkers for immune-mediated diseases (IMIDs), such as rheumatoid arthritis (RA), psoriasis (PS), psoriatic arthritis (PsA), inflammatory bowel disease, and systemic lupus erythematosus (SLE). Our aim was to evaluate plasma Gal1/9 differences between IMID patients and healthy donors (HD). We analyzed 980 plasma samples divided into two analytical cohorts (600 discovery group and 380 validation group). Generalized linear models estimated Gal1/9 levels, adjusting for sex, age, storage time, and plate variability. In the overall IMID group, plasma Gal1 levels were comparable to those of HD, while Gal9 levels were significantly elevated. Levels varied across individual diseases: SLE patients consistently showed the highest Gal1/9 levels compared to both HD and other IMIDs, and RA patients had elevated Gal9 levels versus HD. Both Gal1 and Gal9 plasma levels correlated positively with higher disease activity, and Gal1 was higher in patients with longer disease duration. After adjustment for these confounders, SLE and RA patients maintained the highest Gal9 levels compared to HD. Our study demonstrates that Gal1 and Gal9 are differentially expressed across IMIDs, with particularly elevated levels in SLE, and both galectins are associated with disease activity.

## 1. Introduction

Immune-mediated inflammatory diseases (IMIDs) are a broad group of disorders characterized by abnormal immune responses that lead to inflammation, tissue damage, and, in many cases, long-term complications. Examples of IMIDs are rheumatoid arthritis (RA), psoriasis (PS), psoriatic arthritis (PsA), spondyloarthritis (SpA), inflammatory bowel disease (IBD), and systemic lupus erythematosus (SLE). The prevalence of IMIDs in Western society is around 5–7% [1]. IMIDs are complex, heterogeneous diseases with an etiology that remains unclear, likely due to their molecular complexity and polygenic nature and the presence of environmental risk factors such as smoking, overweight, advanced age, and infections. There is an overlap of risk loci between the different IMIDs, especially for those loci with immune-related genes, suggesting that IMIDs share common signaling pathways [2].

Although multiple factors may interact to promote the development of IMIDs, it is generally believed that most of these disorders arise due to a deficiency in immune tolerance or an imbalance between activation and termination of the immune response [3]. IMIDs share a common hallmark: an immune cell-mediated inflammation that affects various organs and systems [1], resulting in diverse clinical manifestations. However, the presence of overlapping clinical features, especially early in the disease course and in conditions such as inflammatory arthropathies, poses a challenge to precise classification. Additionally, delayed diagnosis in IMIDs is well known to be associated with worse clinical outcomes. In these diseases, the therapeutic goal is to achieve long-term and sustained control of inflammation to prevent tissue damage [4]. As a result, strong evidence in RA [5,6], PsA [7,8], and SpA [9] recommends implementing a treat-to-target strategy and supports the initiation of immunomodulatory therapy as soon as the diagnosis is established. Thus, the identification of additional accurate biomarkers for early diagnosis and poor prognosis remains essential.

Galectins are β-galactoside-binding animal lectins expressed in many tissues and organs, which show their highest expression in the immune system, where they regulate its homeostasis [10,11]. Galectins are characterized by shared consensus amino acid sequences in the carbohydrate-recognition domain (CRD) [10]. The variety of binding partners and the widespread distribution of galectins enable them to participate in multiple biological functions, including the regulation of the immune system [12]. In this regard, galectins play a key role as immune regulators, capable of amplifying or dampening immune and inflammatory responses. These responses depend on factors such as the inflammatory stimulus and the microenvironment or the target cell [13,14]. Emerging literature indicates that galectins are involved in the pathogenic mechanisms and development of IMIDs, including RA, SLE, IBD, or PS [15,16]. These characteristics make these proteins attractive therapeutic targets and potential biomarkers for IMIDs.

Galectin 1 (Gal1) acts as a dual regulator of the immune system, playing protective or anti-inflammatory roles in some contexts (autoimmune diseases, allergies, atherosclerosis) while exacerbating pathological processes in others (cancer, infections, osteoarthritis) [12,16,17]. Gal1 suppresses pro-inflammatory cytokine production; modulates monocyte, macrophage, and dendritic cell activity; limits neutrophil and eosinophil migration; and promotes regulatory T cell function. Through these mechanisms, Gal1 mitigates Th1- and Th17-mediated responses, drives the immune response toward a Th2-type profile, and supports the resolution of inflammation [13,16,17].

Galectin-9 (Gal9), as Gal1, acts as an immunomodulator that regulates both adaptive and innate immune responses. In T cells, Gal9 binds to receptors such as TIM-3, promoting apoptosis of pathogenic Th1/Th17 pathways, expanding Tregs, and therefore limiting effector T cell responses [18]. Conversely, intracellular Gal9 can enhance TCR signaling and support Th17 differentiation, potentially exacerbating autoimmune pathology.

Beyond T cells, Gal9 also inhibits TLR7/TLR9-mediated activation of plasmacytoid dendritic cells and B cells, thereby reducing type I interferon secretion and autoantibody production [19,20].

Both galectins are differentially expressed depending on the inflammatory milieu, thereby influencing leukocyte recruitment, activation, and polarization toward pro- or anti-inflammatory phenotypes [21]. Their pleiotropic actions are highly context- and tissue-dependent, and they are currently being investigated as therapeutic targets for modulating immune responses in multiple IMIDs [13,21]. Our group previously validated that Gal1 levels were elevated in the serum of patients with early RA compared to healthy donors (HD) [22]. In the present study, our objective was to explore whether Gal1 levels are also elevated in other IMIDs, beyond RA, compared to HD, thereby validating our previous findings in RA [22]. Furthermore, Gal9 was included in this study because of its functional similarity to Gal1 and the previously observed increase in serum levels in RA and SLE [21]. These approaches may help clarify whether the elevation of Gal1 and Gal9 reflects a general regulatory mechanism in IMIDs or supports their potential utility as diagnostic biomarkers, particularly in RA.

## 2. Results

### 2.1. Study Population

We analyzed a total of 980 plasma samples divided into two analytical cohorts: 600 samples in the discovery group (group 1) and 380 in the validation group (group 2). Of these, 150 were from healthy donors (HD) and 830 from patients with IMIDs, including 150 with RA, 165 with PsA, 165 with PS, 150 with SLE, 100 with UC, and 100 with CD. The main characteristics of these populations are presented in Table 1. Overall, there were more women in the SLE and RA patient groups, and the sex distribution of these patients was more balanced in group 1. Patients with PsA, and especially those with RA, were older than those in other disease groups, and their age was evenly distributed between group 1 and 2. Samples were collected at baseline diagnosis from 10 patients, while only 55 patients (6.6%) had early-stage disease (<2 years since onset). The majority (*n* = 650/830; 78.3%) exhibited long-standing disease (>5 years of duration), particularly among patients with RA, SLE, and PS. In group 1, patients with PS and CD exhibited longer disease duration compared to those in group 2, while no significant differences were observed for the other IMIDs. Moderate-to-high DA was observed in nearly a quarter of the patients at the time of sample collection (*n* = 191; 23%), with significantly higher activity observed in RA patients (*n* = 77; 41%) compared to those with other IMIDs. Most patients with SLE, RA, and IBD were receiving conventional synthetic Disease-Modifying Antirheumatic Drug therapy, while the use of biological therapy was substantially lower overall. However, it was moderate for RA patients (*n* = 73; 48.6%), exceeding that for other IMIDs.

### 2.2. Gal1 and Gal9 Plasma Levels in Different IMIDs

**Figure 1:**
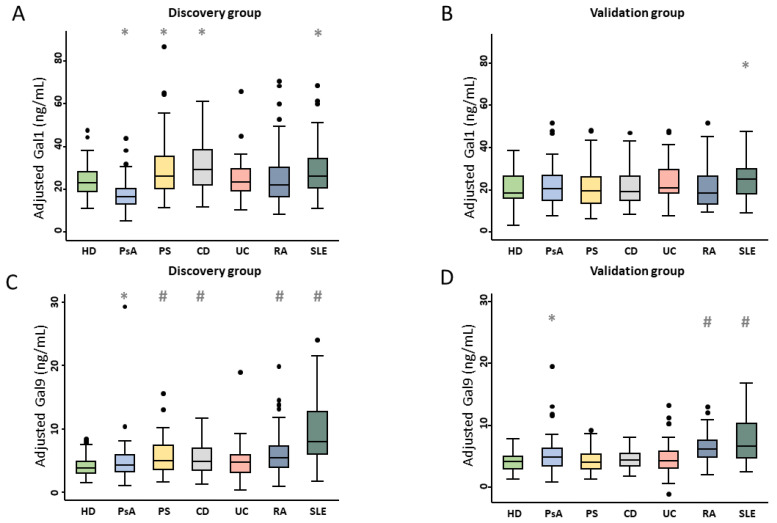
Gal1 and Gal9 plasma levels across IMIDs and HD in the discovery (**A**,**C**) and validation (**B**,**D**) populations. Values of Gal1 and Gal9 plasma levels were adjusted for frozen storage time, age, sex, and inter-assay variability (plate). Data are shown as box plots in which the line in the box represents the median, and the box edges represent the interquartile range. Whiskers represent the 5th and 95th percentiles. Dots indicate outliers. The Mann–Whitney test was used to determine significant differences with respect to HD. Due to multiple comparisons, the level of significance was set at *p* < 0.008 (* *p* > 0.008 to <0.05; # *p* < 0.008). Abbreviations: CD: Crohn’s disease; Gal1: galectin 1; Gal9: galectin 9; HD: healthy donors; IMIDs: immune-mediated inflammatory diseases; PS: psoriasis; PsA: psoriatic arthritis; RA: rheumatoid arthritis. SLE: systemic lupus erythematosus; UC: ulcerative colitis.

Plasma Gal1 levels in the overall IMID group were comparable to those of HD, both in group 1 (22.6 [17.2–31.05] vs. 23.5 [18.9–28.2]; *p* = 0.816) and in group 2 (14.8 [20.5–27.0] vs. 18.4 [16.1–26.4]; *p* = 0.626). In addition, as shown in Figure 1A,B, compared to HD, patients with SLE showed a non-statistically significant increase in Gal1 levels in both groups (*p* = 0.008 group 1 and *p* = 0.03 group 2), and these levels were also higher compared to RA, PsA, UC, and CD in both groups (Supplementary Table S1). In group 1, Gal1 levels were higher in patients with CD and PS compared to HD and lower in those with PsA. They were also greater in PS and RA (*p* < 0.001) than in PsA; however, those findings were not replicated in group 2. In contrast, other IMIDs such as UC and RA did not show significant differences in Gal1 levels vs HD. Among IBD subtypes, CD patients in group 1 exhibited higher Gal1 levels than UC patients (*p* < 0.001), although this difference was not observed in group 2.

Unlike Gal1, plasma Gal9 levels were significantly higher in the overall IMID group compared to HD in both cohorts (group 1 (5.3 [3.6–7.5] vs. 3.8 [2.9–4.8]; *p* < 0.001) and group 2 (4.8 [3.5–6.6] vs. 4.1 [3.0–5.0]; *p* = 0.004), despite considerable overlap in values. When analyzing individual IMIDs, patients with SLE, followed by RA, consistently showed significantly higher Gal9 levels than HD in both groups (*p* < 0.001 for both) (Figure 1C,D). As observed with Gal1, SLE patients exhibited the highest Gal9 levels among all IMIDs studied (Supplementary Table S2).

On the other hand, PsA patients showed only a trend toward increased levels vs. HD (*p* = 0.06 in group 1; *p* = 0.04 in group 2) and had significantly lower levels than RA patients (*p* < 0.001 in group 1; *p* = 0.004 in group 2). Similar to Gal1, Gal9 levels in PsA patients were also lower than in PS patients in group 1, although this difference did not reach statistical significance. Among the other IMIDs, CD and PS patients had significantly higher Gal9 levels than HD only in group 1 (*p* < 0.001), with no significant differences observed in group 2.

### 2.3. Association of Gal1 and Gal9 Plasma Levels with Disease Activity and Disease Duration

Given the broad variability in Gal1 and Gal9 levels across IMIDs, we hypothesized that disease duration or DA could contribute to this heterogeneity. To assess this contribution, DA was standardized into a common categorical variable using disease-specific activity indices to define the categories in each disease, as described in the Methods section.

**Figure 2:**
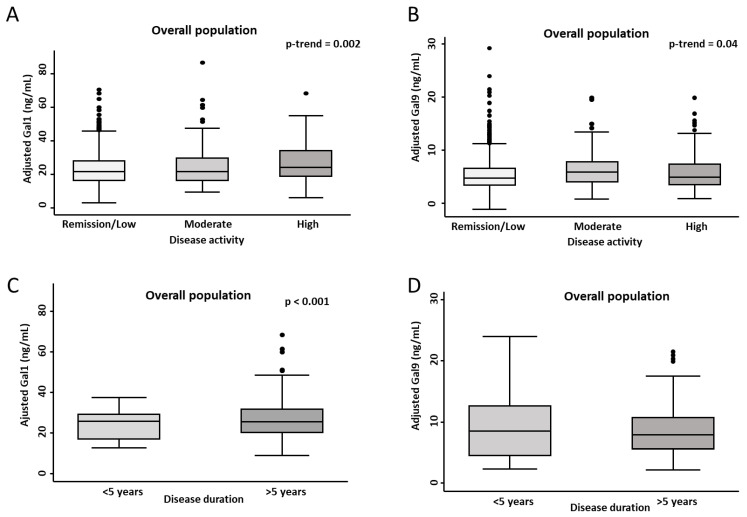
Gal1 and Gal9 plasma levels according to disease activity category (**A**,**B**) and disease duration (**C**,**D**) in patients with immune-mediated inflammatory diseases. Box plots show the interquartile range (IQR; box edges = 25th to 75th percentile, midline = median), and whiskers represent the 5th and 95th percentiles. Dots indicate outliers. Values for Gal1 and Gal9 plasma levels were predicted from multivariable linear regression models adjusted for frozen storage time, age, sex, and inter-assay variability (plate). Statistical significance was determined by Cuzick’s test (panels **A**,**B**) and the Mann–Whitney test (panels **C**,**D**); significance level was set at *p* < 0.05. Abbreviations: CD: Crohn’s disease; Gal1: galectin 1; Gal9: galectin 9.

When analyzing DA in the overall population, we observed a significant positive trend toward higher plasma levels of Gal1 (*p* = 0.002) and Gal9 (*p* = 0.041) across increasing DA categories. This suggests a progressive rise in galectin levels with worsening DA (Figure 2A,B). Among the different IMIDs, patients with moderate and high DA exhibited significantly higher Gal1 levels in RA (*p* = 0.0005), while a non-significant trend was observed in UC (*p* = 0.01) (Supplementary Figure S1). Regarding Gal9, non-significant trends toward elevated levels in patients with increased DA were found in SLE (*p* = 0.0306), PS (*p* = 0.0094), and RA (*p* = 0.0240) (Supplementary Figure S2).

**Figure 3:**
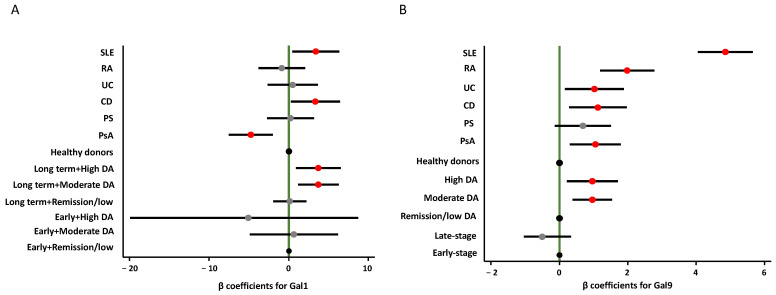
Variables associated with Gal1 and Gal9 plasma levels. Data represent de β coefficients (dots) and the 95% confidence interval (black line) obtained in multivariable linear regression models assessing the association between plasma levels of Gal1 (**A**) and Gal9 (**B**) and the following variables: diagnosis (HD (refence), SLE, RA, UC, CD, PS, PsA), disease activity (high, moderate, remission/low), and disease duration (early vs. late-stage). Black dots represent the reference condition of each variable, gray dots represent non-significant conditions, and red dots significant conditions. (See Supplementary Tables S3 and S4 and Methods section for further information). Abbreviations: CD: Crohn’s disease; DA: disease activity; Gal1: galectin-1; Gal9: galectin-9; PS: psoriasis; PsA: psoriatic arthritis; RA: rheumatoid arthritis. SLE: systemic lupus erythematosus; UC: ulcerative colitis.

Additionally, we compared patients with early-stage (<5 years) and late-stage (≥5 years) IMIDs (Figure 2C,D), detecting significantly higher Gal1 plasma levels in those with longer disease duration (*p* < 0.001). However, this pattern was not evident for Gal9. As shown in Figure 3, the best multivariable model for Gal1, which included diagnosis, DA, and disease duration, revealed a significant interaction between the latter two variables. Notably, only patients with long-standing disease and moderate to high DA exhibited significantly elevated plasma Gal1 levels, compared to early-stage patients in remission or with low DA (Figure 3, left panel and Supplementary Table S3; *p* = 0.004 and *p* = 0.010, respectively). However, among patients with early-stage disease, no relevant association was observed between Gal1 plasma levels and DA. In contrast, Gal9 levels were not significantly influenced by disease duration, but patients with moderate and high DA showed significantly elevated Gal9 plasma levels compared to those in remission or with low DA (Figure 3, right panel, and Supplementary Table S4; *p* = 0.001 and *p* = 0.011, respectively).

After adjusting for DA and stratifying by disease duration, patients with PsA showed significantly lower Gal1 plasma levels (*p* = 0.001), while those with CD (*p* = 0.034) and SLE (*p* = 0.023) exhibited significantly higher levels compared to HD (Figure 3, left panel; Supplementary Table S3). In contrast, all IMIDs except PS displayed elevated Gal9 levels relative to HD (Figure 3, right panel; Supplementary Table S4).

## 3. Discussion

The main strength of this study lies in demonstrating that plasma levels of Gal1 and Gal9 are dysregulated in patients with IMIDs, through an assessment within the same population and across two independent cohorts. Our findings suggest that plasma levels of Gal1 and particularly Gal9 are generally elevated in IMIDs compared to HD, with galectin levels influenced by disease activity in both cases and by disease duration in the case of Gal1. These results reinforce a potential role for galectins in the pathophysiology of IMIDs as key regulators of immune and inflammatory responses [13,14].

It is worth noting that SLE was the only IMID in which both Gal1 and Gal9 levels were consistently elevated compared to HD across both cohorts. Furthermore, these results remained significant after adjustment by disease duration and disease activity. Gal9 was also increased in RA, whereas findings for other IMIDs versus HD were inconsistent between the two study populations. However, after adjustment by disease activity level, all IMIDs except PS showed increased Gal9 plasma levels.

The differential levels of Gal9 and Gal1 observed across IMIDs can be interpreted in light of their dual immunoregulatory roles and the predominant immune pathways in each condition [21]. Both Gal1 and Gal9 share key anti-inflammatory properties at the level of adaptive immunity, particularly by inhibiting Th1/Th17 responses (decreased IFNγ, TNF-α, and IL-17), promoting expansion of Foxp3+ regulatory T cells, and attenuating pathogenic T cell activity [12,13,16,17,19]. However, their effects diverge in humoral and innate immunity. Gal9 inhibits plasmablast differentiation and autoantibody generation, whereas Gal1 favors a Th2-skewed response. At the innate immunity level, Gal1 predominantly exerts anti-inflammatory effects (e.g., neutrophil apoptosis, M2 macrophage polarization), while Gal9 enhances granulocyte survival, activation, and pro-inflammatory cytokine release [12,13,16,17,19,21]. Overall, their anti-inflammatory mechanisms appear to dominate over their pro-inflammatory properties, consistent with the protective effects observed in murine models of SLE and RA, where administration of either galectin ameliorated disease activity [23,24,25,26]. In this context, the particularly high Gal9 levels observed in our SLE and RA patients compared to other IMID patients could reflect its additional inhibitory role in humoral responses, beyond T cell modulation. Conversely, the highest Gal1 levels observed in SLE, but not in other IMIDs, may be interpreted as a consequence of its anti-inflammatory function in the innate immunity, which plays a central role in SLE pathogenesis.

To the best of our knowledge, our study provides the first description in the literature of plasma galectin levels across all these IMIDs within the same population. Previous studies focused on individual IMIDs had already reported increased plasma levels of Gal1 in patients compared to controls in RA [21,22], IBD [27,28], and PS [29]. However, no evidence on Gal1 levels was available for SLE or PsA. Regarding Gal9, other publications had also reported elevated plasma levels in patients with RA [30] and SLE [31,32] compared to controls, in accordance with our study. In PS, Chagan-Yatusan et al. [33] found elevated levels of Gal9 compared to HD, whereas Nofal et al. [34] described similar Gal9 levels, in line with our results. In contrast, our PsA patients had a trend to higher Gal9 levels than the controls. In this case, no previous studies had assessed Gal9 levels in PsA compared to HD. In IBD, a study found no association between higher Gal9 levels and the disease [35], and similarly, in our cohort, elevated Gal9 levels were only observed in patients with IBD after adjusting for disease activity.

Once it was established that these galectins could be elevated in certain IMIDs, our study aimed to identify differences between some of them. Remarkably, the highest levels of both Gal1 and, especially, Gal9 were observed in SLE patients compared to those with other IMIDs, a finding that was replicated in both cohorts.

Interestingly, PsA patients exhibited significantly lower Gal9 levels compared to RA patients in both cohorts; however, lower Gal1 levels were observed in the discovery cohort, but this result was not replicated in the validation cohort. Previous evidence from our group had suggested that Gal1 levels in both serum and synovial fluid were higher in early RA compared to SpA [36]. More recently, and in line with our findings, Bably et al. [37] reported higher serum Gal1 levels in RA compared to PS and PsA, with no differences between PS and PsA. In our study, Gal1 and Gal9 levels were lower in PsA than in PS; although this finding could not be validated in both cohorts. No reports were found in the literature comparing Gal9 levels between PsA and PS; however, Gal9 has been investigated in eczematous inflammatory skin conditions, showing elevated levels in atopic dermatitis compared to PS [34].

Although we cannot draw firm conclusions at this stage, these findings may be useful in distinguishing certain clinical patterns in some IMIDS—for example, in cases of undifferentiated arthritis, where SLE, RA, PsA, and osteoarthritis should be considered in the differential diagnosis. In our analysis in IBD, we were also unable to establish consistent differences in Gal1 or Gal9 levels between CD and UC. Previous studies in IBD have reported no differences in Gal1 and Gal9 plasma levels between CD and UC [27,38], nor in the mRNA expression of Galectins-1, -2, -3, -4, and -9 [39].

Given the anti-inflammatory properties of Gal1 and Gal9 [12,13,14,15,16], their elevated levels in IMIDs may represent a mixed regulatory loop mechanism aimed at controlling or promoting inflammation, which could be associated with disease activity or severity. Supporting this interpretation, our study revealed that Gal9 plasma levels were increased in patients with moderate or high DA compared to those in remission or low DA. Regarding Gal1, this association could only be demonstrated in late-stage patients. Interestingly, in RA patients, Mendez-Huergo et al. [40], in a population mostly including long-standing RA, reported correlations between Gal1 plasma levels and DA assessed with both the ESR and the DAS-28 indices. Conversely, our previous observations from longitudinal studies on early RA revealed no clear correlation between serum Gal1 levels and DA [22], in agreement with results reported by Bably et al. [37]. Altogether, these results suggest that increased Gal1 levels could be associated with severity in long-term RA patients rather than being a disease activity biomarker in early arthritis. Considering Gal9 in RA, substantial evidence has shown that its plasma levels positively correlate with increased disease activity and severity, as reflected by higher disease scores, elevated acute-phase reactants, greater treatment burden, and longer disease duration [30,41,42,43,44,45]. Moreover, Gal9 levels have been shown to decrease in response to effective immunosuppressive therapy [43,46].

In the case of SLE, our data confirmed an association between Gal9 plasma levels (not confirmed for Gal1) and higher DA, consistent with previous studies using the SLEDAI-2K index or the type I IFN gene signature [21,47,48,49,50,51]. These studies also reported correlations with disease severity—reflected by organ involvement and damage—and with treatment response, as Gal9 levels decreased with clinical improvement. On the contrary, urinary Gal-9 levels do not differentiate active from inactive lupus and are similar to those of controls [47,48].

As for SLE, patients with PS exhibited a trend to higher plasma Gal9 levels correlating with PS activity, which were not observed for Gal1; however, other studies found no differences in Gal1 or Gal9 levels [29,34]. Our cohort exhibited heterogeneous results in the association between IBD and disease activity, and the limited number of patients in the moderate-to-high activity subgroup precludes drawing definitive conclusions. Previous studies have reported a correlation of IBD flares and histological colonic inflammation with both serum levels and mRNA expression of Gal1 [27,38,52], whereas others have found no significant differences in Gal1 serum levels between active disease and remission [27]. As for Gal9, Cibor et al. [35] found no association between its serum levels and disease activity in either CD or UC.

Some limitations of this study must be considered: (a) the cross-sectional design does not allow us to determine whether galectin levels vary over time during patient follow-up, nor does it allow us to confirm whether Gal1 remains stable over time in other IMIDs, as previously observed in RA and SpA [22,36]; (b) there is a lack of clinical and therapeutic data, such as obesity, cardiovascular disease, and cancer, which have been associated with elevated galectin levels [53,54,55,56] and could improve adjustment of Gal1 and Gal9 plasma levels; (c) the study population was limited to Caucasian individuals, which may affect the generalizability of Gal1 and Gal9 as potential biomarkers for IMIDs. For instance, in Gal9 studies, results reported in Asian cohorts were similar to those obtained in our study, both in RA [30,41,42,43,46] and in SLE [31]. In SLE, our Gal9 results have been also replicated in other different cohorts (Egyptian, Indian, or Turkish [32,48,49]); (d) the use of a unified categorical disease activity variable across diverse IMIDs. While widely accepted cut-offs were applied for each index, the constructs differ substantially (e.g., joint counts, skin involvement, serology), so “moderate” or “high” activity may not reflect equivalent inflammatory burden. This common construct may partly explain the heterogeneity across activity categories, but it enabled the inclusion of all IMIDs and provided sufficient power to detect differences related to disease activity; (e) most patients had long-standing disease (>10 years), and the lower proportion with early disease may have limited the sensitivity and statistical power to detect associations, despite applying a 5-year cut-off to distinguish early from later disease; finally, we cannot rule out the influence of the smaller sample size in the validation cohort, which may have limited the ability to detect subtle differences in galectin levels.

## 4. Methods

### 4.1. Patients and Samples

In this study, we requested plasma samples from patients diagnosed with six different IMIDs, including RA, PsA, SLE, IBD (ulcerative colitis [UC], Crohn’s disease [CD]), and PS, as well as HD from different sources. The samples from 830 patients were provided by Hospital Universitario Vall d’Hebron Biobank, collected by the Immune Mediated Inflammatory Disease Consortium (IMIDC) between June 2007 and December 2010. HD samples from 150 individuals were collected from IIS-IP Biobank and Spanish National DNA Bank Carlos III (Salamanca University). Samples were collected during patient follow-up after the initial diagnosis and were sequentially assigned to one of two groups based on their order of arrival at our center: a discovery group (1) and a subsequent validation group (2). Galectin determination was conducted in batches at two different time points in each group (2022 and 2024). We included patients with a confirmed diagnosis of a single IMID, excluding those with more than one IMID. Diagnostic and inclusion criteria for each IMID were as follows:

- RA: American College of Rheumatology (ACR) classification criteria for RA [57].
- PsA: Classification Criteria for Psoriatic Arthritis (CASPAR) [58].
- PS: diagnosis based on the dermatologist clinical criteria.
- IBD: diagnosed according to the standard Lennard-Jones criteria for CD and UC [59].
- SLE: 1982 revised ACR criteria for SLE classification [60].

### 4.2. Measurement of Gal1 and Gal9 Plasma Levels in HD and IMID Population

Gal1 and Gal9 were measured in plasma samples from all patients and HD by using the Human Galectin-1 Quantikine ELISA Kit DGAL10 (R&D Systems; Minneapolis, MN, USA), and the Human Galectin-9 Quantikine ELISA Kit DGAL90 (R&D Systems; Minneapolis, MN, USA) according to the manufacturer’s instructions. All samples were tested in duplicate in each plate, and, to minimize the influence of inter-assay variability, samples from the different IMIDs were included in every plate. Once the procedure for each ELISA was performed, plates were immediately read in a spectrophotometer (Innogenetics Diagnóstica y Terapéutica S.A.U, Barcelona, Spain) Absorbance was measured at 450 nm with correction at 620 nm.

### 4.3. Statistical Analysis

The time of sample collection; demographic, laboratory, and disease-related data; and treatment patterns were provided by Hospital Universitario Vall d’Hebron Biobank. Differences in demographic and clinical characteristics between the discovery and validation populations, as well as across different diseases, were analyzed. For plasma levels of Gal1 and Gal9, their variations across different IMID diagnoses and their relationship with disease duration and moderate or high disease activity (DA) were analyzed. To allow comparative analyses across the cohort, a unified DA variable was created, classifying each patient into one of three activity levels (remission/low, moderate, and high DA). DA was assessed using validated indices specific to each condition, applying the following cut-off values to categorize remission/low, moderate, and high DA, respectively: DAS-28 ≥ 3.2 and ≥5.1 for PsA and RA [61]; PASI ≥ 7 and ≥15 for PS [62]; SLEDAI ≥ 6 and ≥10 for SLE [63]; Harvey–Bradshaw Index ≥ 8 and ≥16 for CD [64], and Lichtiger score ≥ 9 and ≥15 for UC [65]. To avoid missing data at multivariable analysis exploring factors that can impact DA, HD were included in the remission/low activity group.

Most quantitative variables showed a non-normal distribution and were therefore presented as median and interquartile range (IQR). The Mann–Whitney U test or Kruskal–Wallis test was used to assess significant differences in Gal1 and Gal9 plasma levels according to clinical or laboratory variables and across different diagnostic groups. Bonferroni correction was applied to adjust for multiple comparisons, with *p*-values considered statistically significant if below 0.008. Correlations between continuous variables were analyzed using Spearman’s rho test. In a previous study, we demonstrated that Gal1 levels can be influenced by sample storage time, age, sex, and inter-assay variability (plate) [22]. Accordingly, generalized linear models (GLMs) were independently fitted for Gal1 and Gal9 within each group, using the respective galectin as the dependent variable and including the aforementioned covariates to obtain more accurate adjusted estimates. GLMs were used to evaluate differences between IMID diagnosis and HD or other IMIDs, DA, or duration of disease. Forest plots were generated to visualize the associations of Gal1 and Gal9 with DA, incorporating interaction terms to assess the modifying effect of disease duration. Statistical analyses were performed using Stata 14.0 for Windows (StataCorp LP, College Station, TX, USA).

## 5. Conclusions

Our study revealed that plasma levels of Gal1 and Gal9 are differentially regulated across various IMIDs, with particularly elevated levels observed in SLE patients compared to other IMIDs. Additionally, Gal1 and Gal9 may serve as biomarkers for monitoring disease activity, with Gal1 potentially influenced by disease duration. While our data validate the utility of Gal9 in RA and SLE, we also highlight the need to further explore the role of Gal1 in both diseases.

## Figures and Tables

**Table 1:** Characteristics of patients diagnosed with IMIDs and controls.

Diagnosis	Total Patients (*n* = 830)	Age (Median; IQR)	Sex (Female) (*n*; %)	Duration of Disease (Years; Median: IQR)	Moderate/ High Disease Activity ** (*n*; %)	csDMARD (*n*; %)	bDMARD (*n*; %)
HD	150	44 (30–75)	87 (58)				
Group 1	100	35 (28–48)	57 (57)				
Group 2	50	48 (44–53)	30 (60)				
PsA	165	52.5 (42.5–63)	85 (51.5)	10 (5–17)	42 (25.4)	92 (55.7)	41 (24.8)
Group 1	100	50 (42–63)	50 (50)	11 (5–17)	22 (22)	56 (56)	31 (31)
Group 2	65	54 (43–61)	35 (53.8)	10 (5–16)	20 (30.7)	36 (65.4)	10 (15.3)
Psoriasis	165	44.5 (33–59)	82 (49.6)	15 (6–25)	39 (23.6)	31 (18.2)	42 (25.4)
Group 1	100	49 (37–60)	50 (50)	17.5 (9–27.5)	24 (24)	26 (26)	33 (33)
Group 2	65	40 (28–53)	32 (49.2)	11 (4–22)	15 (23)	5 (7.6)	9 (13.8)
CD	100	39 (33–49)	46 (46)	12 (6–18)	6 (6)	79 (79)	11 (11)
Group 1	50	42 (36–52)	25 (50)	16.5 (11–21)	4 (8)	38 (76)	11 (22)
Group 2	50	36 (30–44)	21 (42)	7.5 (4–13)	2 (4)	41 (82)	0 (0)
UC	100	43.5 (35–57.5)	51 (51)	10 (7–16)	4 (4)	72 (72)	7 (7)
Group 1	50	39.5 (32–53)	25 (50)	10 (7–14)	4 (4)	42 (84)	3 (6)
Group 2	50	50.5 (39–60)	26 (52)	12 (8–17)	0 (0)	30 (60)	4 (8)
RA/sero+ *	150/62	63 (54–71)	90 (60)	13 (8–19)	77 (41)	105 (70)	73 (48.6)
Group 1	100	62 (54–71)	50 (50)	13 (8–18)	53 (53)	88 (88)	63 (63)
Group 2	50	64 (56–72)	40 (80)	12.5 (8–23)	24 (48)	14 (28)	10 (20)
SLE	150	44 (33–52)	99 (66)	14 (8–19)	23 (15.3)	97 (64.6)	4 (4.6)
Group 1	100	42.5 (32–51)	50 (50)	13 (7.5–18)	17 (17)	62 (62)	3 (3)
Group 2	50	47 (38–52)	49 (98)	15.5 (9–21)	6 (12)	35 (70)	1 (2)

Group 1: discovery cohort; Group 2: validation cohort. * Sero+: seropositive for both rheumatoid factor and anti-citrullinated peptide antibodies. ** Moderate-high disease activity was evaluated from assessments with and specific index for each IMID according to the following cut-offs: DAS-28 > 3.2 for PsA and RA, PASI > 10 for PS, SLEDAI > 6 in SLE, Harvey-Bradshaw > 8 in Crohn’s disease, and Lichtiger score > 8 in ulcerative colitis. Abbreviations: CD: Crohn’s disease; bDMARD: biological disease-modifying antirheumatic drug; csDMARD: conventional synthetic DMARD; HD: healthy donors; IQR: interquartile range; n: number; PsA: psoriatic arthritis; RA: rheumatoid arthritis; SLE: systemic lupus erythematosus; UC: ulcerative colitis.

## Data Availability

The original contributions presented in this study are included in the article/Supplementary Material. Further inquiries can be directed to the corresponding authors.

## Supplementary Materials

The following supporting information can be downloaded at https://www.mdpi.com/article/10.3390/ijms26189087/s1.

## Abbreviations

The following abbreviations are used in this manuscript:

CD	Crohn’s disease
DA	disease activity
DAS-28	disease activity score 28 (joints)
ELISA	enzyme-linked immunosorbent assay
GLMs	generalized linear models
HD	healthy donors
IBD	inflammatory bowel disease
IMIDs	immune-mediated inflammatory diseases
PASI	Psoriasis Area and Severity Index
PsA	psoriatic arthritis
PS	psoriasis
RA	rheumatoid arthritis
SLE	systemic lupus erythematosus
SLEDAI	Systemic Lupus Erythematosus Disease Activity Index
UC	ulcerative colitis
