# Supplementary material for: Differential Expression of Galectin-1 and Galectin-9 in Immune-Mediated Inflammatory Diseases
Source: Int J Mol Sci. 2025 Sep 18;26(18):9087. doi: 10.3390/ijms26189087 (PMC12470268; doi:10.3390/ijms26189087)
Supplement: Supplementary file 1 [file ijms-26-09087-s001.zip › ijms-3841989-supplementary.pdf]

International Journal of
Molecular Sciences

MDPI

Supplementary Material

Supplementary Tables

Supplementary Table S1. Galectin-1 levels across IMIDs and HD, with pairwise group comparisons.

Subgroup		Galectin-1 levels (ng/mL)	P-value of each comparison					
		P50 (p25-p75)	PsA	PS	CD	UC	RA	SLE
HD	G1	23.50 (18.91-28.27)	<0.001	0.005	0.0001	0.632	0.652	0.008
	G2	18.4 (16.16-26.43)	0.870	0.557	0.777	0.161	0.366	0.030
PsA	G1	16.45 (13.22-20.44)	//	<0.001	<0.001	<0.001	<0.001	<0.001
	G2	20.48 (14.93-26.9)	//	0.443	0.937	0.378	0.315	0.050
PS	G1	26.40 (20.38-35.26)	<0.001	//	0.111	0.064	0.006	0.374
	G2	19.41 (13.60-26.22)	0.443	//	0.502	0.165	0.830	0.012
CD	G1	29.13 (22.22-38-47)	<0.001	0.111	//	0.001	<0.001	0.056
	G2	19.39 (15.13-26-45)	0.937	0.502	//	0.408	0.366	0.054
UC	G1	23.38 (19.48-29.55)	<0.001	0.064	0.001	//	0.456	0.091
	G2	21.01 (18.36-29.65)	0.378	0.165	0.408	//	0.073	0.314
RA	G1	22.21 (16.62.30.14)	<0.001	0.006	<0.001	0.456	//	0.013
	G2	18.29 (13.33-26.65)	0.315	0.830	0.366	0.073	//	0.006
SLE	G1	26.26 (20.58-34.26)	<0.001	0.374	0.056	0.091	0.013	//
	G2	25.07 (18.20-30.05)	0.050	0.012	0.054	0.314	0.006	//

Median (P50) and interquartile range (p25–p75) of Galectin-1 levels (ng/mL) are shown for each subgroup. Comparison of adjusted Galectin-1 levels between diagnostic groups, using the Wilcoxon rank-sum test. Galectin-1 levels were previously adjusted using generalized linear models for sex, age, storage time, and plate variability. P-values were corrected for multiple comparisons using the Bonferroni method and significant associations ($p < 0.008$) are highlighted in red.

Abbreviations: CD: Crohn disease; G1: group 1 (discovery cohort); G2: group 2 (validation cohort); HD: healthy donors; IMIDs: immune-mediated inflammatory diseases; PS: psoriasis; PsA: psoriatic arthritis; RA: rheumatoid arthritis. SLE: Systemic Lupus Erythematosus; UC: ulcerative colitis.

Supplementary Table S2. Galectin-9 levels across IMIDs and HD, with pairwise group comparisons.

Subgroup		Galectin-9 levels (ng/mL)	P-value of each comparison					
		P50 (p25-p75)	PsA	PS	CD	UC	RA	SLE
HD	G1	3.88 (2.99-4.83)	0.068	<0.001	0.001	0.081	<0.001	<0.001
	G2	4.13 (3.00-5.01)	0.047	0.937	0.525	0.352	<0.001	<0.001
PsA	G1	4.29 (3.26-5.93)	//	0.010	0.087	0.908	<0.001	<0.001
	G2	4.84 (3.50-6.28)	//	0.073	0.137	0.349	0.004	<0.001
PS	G1	5.00 (3.63-7.39)	0.010	//	0.722	0.072	0.371	<0.001
	G2	4.04 (2.99-5.30)	0.073	//	0.568	0.459	<0.001	<0.001
CD	G1	4.92 (3.49-6.94)	0.087	0.722	//	0.230	0.294	<0.001
	G2	4.37 (3.37-5.47)	0.137	0.568	//	0.761	<0.001	<0.001
UC	G1	4.72 (3.08-5.92)	0.908	0.072	0.230	//	0.015	<0.001

	**G2**	4.27 (3.08-5.83)	0.349	0.459	0.761	//	<0.001	<0.001
**RA**	**G1**	5.49 (3.9-7.29)	<0.001	0.371	0.294	0.015	//	<0.001
	**G2**	6.18 (4.81- 7.63)	0.004	<0.001	<0.001	<0.001	//	0.075
**SLE**	**G1**	8.03 (6.00-12.79)	<0.001	<0.001	<0.001	<0.001	<0.001	//
	**G2**	6.66 (4.71-10.31)	<0.001	<0.001	<0.001	<0.001	0.075	//

Median (P50) and interquartile range (p25–p75) of Galectin-1 levels (ng/mL) are shown for each subgroup. Comparison of adjusted Galectin-9 levels between diagnostic groups, using the Wilcoxon rank-sum test. Galectin-9 levels were previously adjusted using generalized linear models for sex, age, storage time, and plate variability. P-values were corrected for multiple comparisons using the Bonferroni method and significant associations ($p < 0.008$) are highlighted in red.
Abbreviations: CD: Crohn disease; G1: group 1 (discovery cohort); G2: group 2 (validation cohort); HD: healthy donors; IMIDs: immune-mediated inflammatory diseases; PS: psoriasis; PsA: psoriatic arthritis; RA: rheumatoid arthritis. SLE: Systemic Lupus Erythematosus; UC: ulcerative colitis.

Supplementary Table S3. Variables independently associated with plasma Galectin-1 levels in the multivariable model.

	**β coefficient**	**CI 95%**	**p**
Early-stage & Remission/Low DA	Ref.	-	-
Early-stage & Moderate DA	0.657	-4.877 – 6.192	n.s.
Early-stage & High DA	-5.066	-19.937 – 8.725	n.s.
Late-stage & Remission/Low DA	0.132	-1.961 – 2.224	n.s.
Late-stage & Moderate DA	3.735	1.182 – 6.289	0.004
Late-stage & High DA	3.720	0.894 – 6.545	0.010
Healthy donors	Ref.	-	-
Psoriatic arthritis	-4.756	-7.534 – -1.977	0.001
Psoriasis	0.225	-2.745 – 3.195	n.s.
Crohn disease	3.352	0.253 – 6.452	0.034
Ulcerative colitis	0.522	-2.639 – 3.682	n.s.
Rheumatoid arthritis	-0.870	-3.804 – 2.064	n.s.
Systemic lupus erythematosus	3.418	0.465 – 6.371	0.023

Beta coefficients (β), 95% confidence intervals (CI), and p-values are shown. The model includes disease stage (early vs. late), disease activity (remission/low, moderate, high), and diagnosis. "Ref." indicates the reference category. n.s. = not significant ($p > 0.05$).
Abbreviations: CI: confidence interval; DA: disease activity; p: p-value

Supplementary Table S4. Variables independently associated with plasma Galectin-9 levels in the multivariable model.

	**β coefficient**	**IC 95%**	**p**
Early-stage	Ref.	-	-
Late-stage	-0.502	-1.038 – 0.034	n.s.
Remission/Low DA	Ref.	-	-
Moderate DA	0.962	0.385 – 1.538	0.001
High DA	0.966	0.221 – 1.713	0.011
Healthy donors	Ref.	-	-
Psoriatic arthritis	1.052	0.304 – 1.799	0.006
Psoriasis	0.687	-0.138 – 1.512	n.s.
Crohn disease	1.125	0.279 – 1.970	0.009
Ulcerative colitis	1.024	0.162 – 1.886	0.020
Rheumatoid arthritis	1.983	1.187 – 2.779	<0.001
Systemic lupus erythematosus	4.856	4.052 – 5.660	<0.001

Beta coefficients (β), 95% confidence intervals (CI), and p-values are shown. The model includes disease stage (early vs. late), disease activity (remission/low, moderate, high), and diagnosis. "Ref." indicates the reference category. n.s. = not significant ($p > 0.05$). Abbreviations: DA: disease activity; CI: confidence interval; p: p-value

Supplementary Figures.

Supplementary Figure S1. Galectin-1 plasma levels across different IMIDs stratified by disease activity.

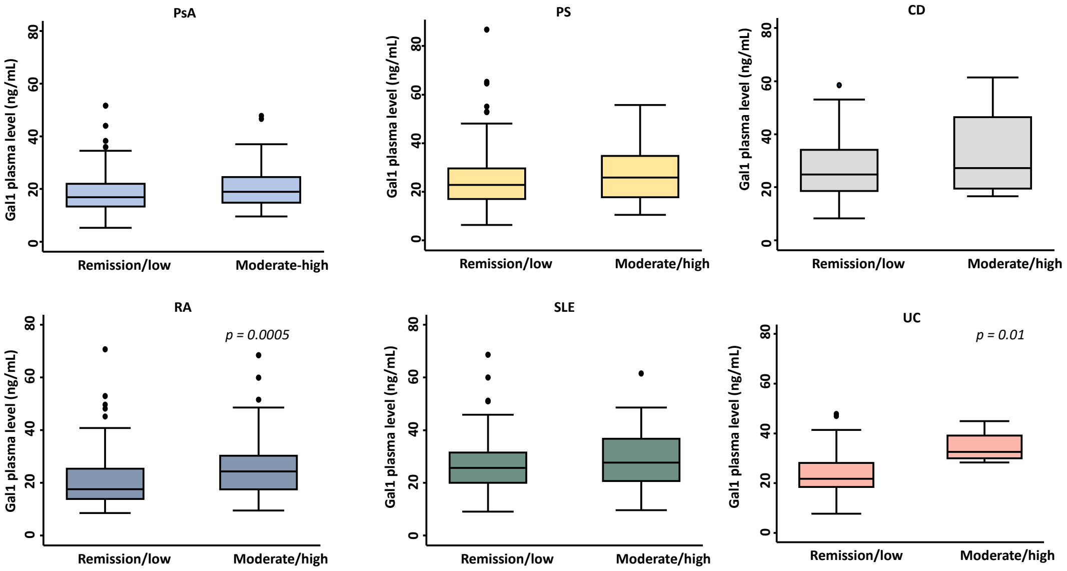

Box plots show the interquartile range (IQR; box edges = 25th to 75th percentile, midline = median), and whiskers represent the 5th and 95th percentiles. Dots indicate outliers. The Mann-Whitney test was used to determine significant differences between groups; Significance was set at $p<0.008$ due to multiple comparisons.

Supplementary Figure S2. Galectin-9 plasma levels across different IMIDs stratified by disease activity

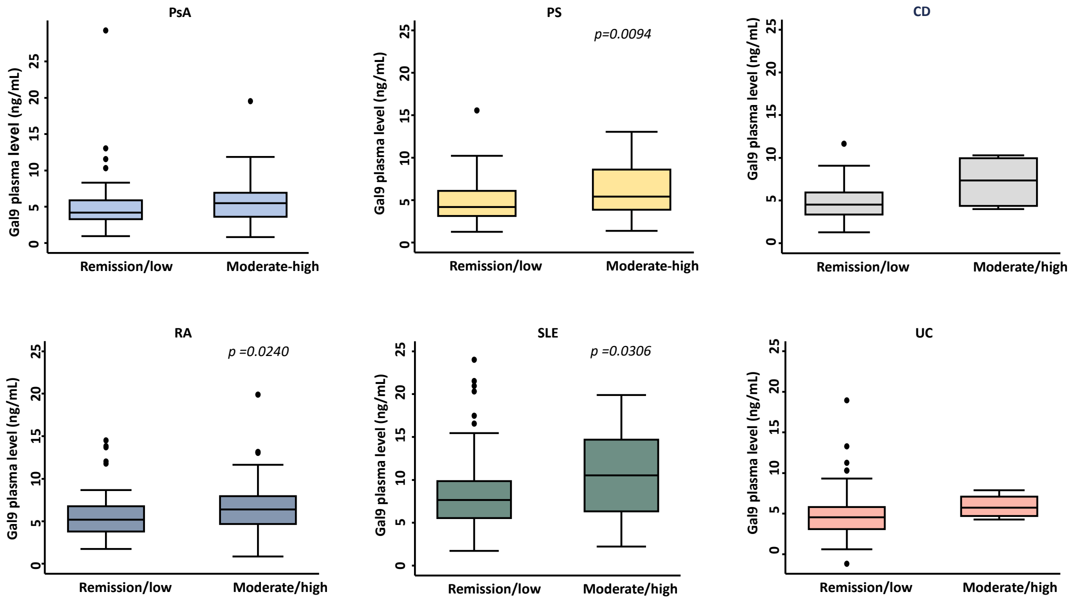

Box plots show the interquartile range (IQR; box edges = 25th to 75th percentile, midline = median), and whiskers represent the 5th and 95th

percentiles. Dots indicate outliers. The Mann-Whitney test was used to determine significant differences between groups; Significance was set at $p<0.008$ due to multiple comparisons.
